# Within-subject foot motion variability in patients with Rheumatoid Arthritis

**DOI:** 10.1186/1757-1146-3-S1-P7

**Published:** 2010-12-20

**Authors:** Lindsey Hooper, Lucy Gates, Lyndsey Goulston, Cathy Bowen, Christopher Edwards, Nigel Arden

**Affiliations:** 1 University of Southampton, Southampton, UK; 2 University of Oxford, Oxford, UK

## Introduction

Multi-segment three-dimensional analysis is a complex yet rapidly evolving methodology in podiatric mechanical research. The purpose of this study was to explore the within-subject foot motion variability (MoVa) during the stance phase of gait.

## Methods

A 3D motion analysis system was used to collect gait data for 5 healthy participants and 5 patients with RA. The oxford foot model was used to characterise dynamic foot & ankle kinematics and spatio-temporal parameters. Inter-segmental motions of interest were defined as tibia-rearfoot and rearfoot-forefoot. The main outcome of interest was within-subject MoVa, expressed as mean standard deviation (SD).

## Results

MoVa ranged from 0.94- 2.33SD and was similar for both groups. Increased MoVa was largely accounted for by rearfoot variance. There is a trend towards increased forefoot MoVa in the RA group (RA 0.65-1.78, Control 0.34-0.9). No single episode during the stance phase had greater variability than any other.

## Discussion

MoVa rather than a particular gait event may be an alternative outcome warranting further investigation. These results suggest care should be taken when assuming mean within-subject MoVa in mechanical analyses. This preliminary work suggests that greater forefoot MoVa may occur in RA participants. This pilot investigation provides useful preliminary data to inform future studies.

